# Corneal and Intraocular Pressure Responses to Scleral Lens Wear: A Meta-Analysis

**DOI:** 10.1007/s44402-026-00110-7

**Published:** 2026-06-02

**Authors:** Clara Martinez-Perez, María Carmen Sánchez-González, José-María Sánchez-González

**Affiliations:** 1 Applied Physics Department (Optometry Area), Facultade de Óptica e Optometría, Universidade de Santiago de Compostela, Santiago de Compostela, Spain; 2 Department of Physics of Condensed Matter, Optics Area, Pharmacy Faculty, University of Seville, Seville, Spain

**Keywords:** Central corneal thickness, Corneal oedema, Contact lens complication, Intraocular pressure, Oxygen permeability, Scleral lenses

## Abstract

**Purpose:**

To evaluate the effects of scleral contact lens wear on central corneal thickness, corneal or stromal swelling and intraocular pressure, and to identify factors that may influence these outcomes.

**Methods:**

A systematic review and meta-analysis was conducted according to the Preferred Reporting Items for Systematic Reviews and Meta-Analyses and the AMSTAR-2 quality assessment tool (registration number PROSPERO CRD420251141392). The PubMed, Web of Science and Scopus databases were searched without language or date restrictions. Eligible studies included prospective, observational, controlled or crossover designs assessing physiological changes during or after scleral contact lens wear. Mean differences with 95% confidence intervals (CIs) were pooled using random- or fixed-effects models. Heterogeneity was quantified using the I-squared statistic, and meta-regressions examined the influence of lens and patient-level factors.

**Results:**

Twenty-two studies, including 830 eyes, were analysed. Scleral contact lens wear produced a small but statistically significant increase in central corneal thickness while the lens was in place (mean difference: 7.93 µm; 95% CI: 4.92–10.95; *p* < 0.001; *I*² = 0%) and no significant change after lens removal (mean difference: 1.49 µm; *p* = 0.34). Corneal or stromal swelling showed a small increase of 0.88% (*p* < 0.001; *I*² = 83%), consistent with the small magnitude and variability of these changes across studies. Intraocular pressure after lens removal showed no significant variation (mean difference: 0.38 mmHg; *p* = 0.27; *I*² = 78%).

**Conclusions:**

Scleral contact lens wear induces minimal and largely reversible changes in corneal thickness and intraocular pressure. Daytime wear of modern high–oxygen-permeable lenses appears to be physiologically safe, although selective monitoring remains advisable in high-risk patients.

Key Points

- Scleral lens wear causes a small, reversible increase in central corneal thickness.
- Corneal and stromal swelling during scleral contact lens wear is minimal and clinically irrelevant.
- Intraocular pressure is not significantly affected by scleral lens wear.

## Introduction

Scleral contact lenses (ScCL) are large-diameter rigid gas-permeable lenses that rest on the sclera and vault the cornea, creating a fluid reservoir between the lens and the corneal surface [1, 2]. Historically reserved for severely compromised eyes, their use has now expanded to include visual rehabilitation in patients with irregular corneas (such as keratoconus, keratoglobus or post-keratoplasty) and the management of severe ocular surface disorders, including Sjögren’s syndrome, exposure keratopathy and Stevens–Johnson syndrome [3–5]. In addition to improving optical quality by neutralising irregular astigmatism, ScCL provide continuous corneal hydration and patients generally report greater comfort and centration compared to corneal rigid gas-permeable lenses [1, 3]. Despite these clinical benefits, scleral lenses can alter corneal physiology due to limited tear exchange and the presence of a post-lens tear reservoir. Although modern designs employ highly oxygen-permeable materials, oxygen must diffuse through both the lens and the fluid layer, reducing its availability to the cornea and potentially leading to hypoxia-related complications such as corneal oedema [2, 6].

Corneal swelling occurs when oxygen supply is insufficient to meet metabolic demands, leading to anaerobic metabolism, lactate accumulation and fluid influx into the stroma [7]. The clinical consequences of hypoxia-induced swelling have long been recognised: lenses made from oxygen-impermeable materials such as polymethyl methacrylate (PMMA) produced striae at 6–8% swelling and visible corneal clouding above 5% [8, 9]. Chronic hypoxia with PMMA has also been linked to endothelial polymegathism and reduced corneal deswelling capacity [10]. These risks motivated the development of high-Dk materials, yet even with modern scleral lenses, corneal oedema remains a concern under both open- and closed-eye conditions.

Experimental studies and theoretical models have investigated how lens and reservoir parameters influence oxygen transmission. Increasing the Dk of scleral materials above 100 or reducing lens thickness has shown limited benefit in decreasing corneal oedema, suggesting that reservoir thickness is the primary limiting factor [11, 12]. Indeed, Fisher et al. [13] demonstrated that reducing the central fluid reservoir from approximately 500 to 150 µm reduced open-eye central corneal oedema by 62%. Under closed-eye conditions, oedema is more pronounced due to lower oxygen tension behind the eyelid. Overnight scleral lens wear has been associated with central corneal swelling ranging between 5 and 17%, with variability linked to endothelial cell function and lens parameters [14, 15]. However, controlled studies under these conditions are scarce, and discrepancies remain regarding the magnitude and clinical relevance of scleral lens-induced oedema.

Adding to the complexity, reports on central corneal thickness (CCT) changes during scleral lens wear are inconsistent. Some studies have found significant increases in CCT after hours or days of wear, particularly in patients with keratoconus [16, 17], while others have described thinning rather than swelling [18–20]. These divergent results highlight the influence of individual ocular physiology, lens design and measurement protocols. Importantly, fluctuations in corneal thickness or oedema, even if small, may compromise visual quality or corneal health if sustained over time [21].

In addition to corneal oedema, the potential effect of scleral lens wear on intraocular pressure (IOP) has generated significant debate. IOP is regulated by the balance between aqueous humour production and outflow within the anterior segment [22]. Any factor that impedes outflow can elevate IOP, and scleral lenses have been hypothesised to do so by compressing episcleral tissues or creating sub-atmospheric pressure beneath the lens [23, 24]. Historical evidence is striking: in 1951, Huggert reported IOP elevations up to 30 mmHg after only 25 min of glass scleral lens wear [25]. However, more recent studies provide mixed results, with some showing transient increases and others reporting no clinically significant changes. The discrepancy likely arises from differences in lens diameter, vault, fitting strategies and tonometric methods.

Taken together, scleral lenses represent a powerful therapeutic and optical tool, yet their physiological impact remains uncertain. While hypoxia-related oedema and potential IOP elevation are recognised concerns, published findings are inconsistent and often limited by small sample sizes, heterogeneous populations and methodological variability, including differences in lens diameter, vault, fitting approach, measurement technique and underlying ocular condition.

Therefore, a systematic review and meta-analysis was conducted to synthesise the available evidence, quantify the magnitude of physiological changes and explore potential sources of variability related to lens design, vault, oxygen transmissibility, patient characteristics and study methodology. Rather than attempting to resolve existing controversies, the review aims to clarify the current state of evidence and identify key gaps to inform future research and clinical practice.

## Methods

### Research Question and Population, Intervention, Comparison, Outcome, Study Design (PICOS) Framework

**Fig. 1:**
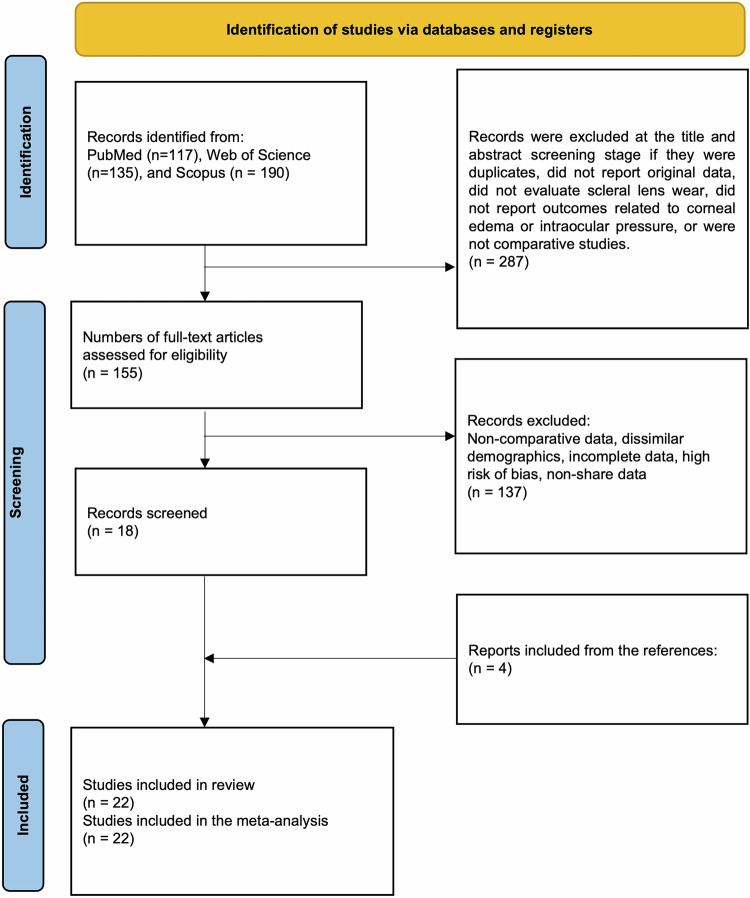
Preferred Reporting Items for Systematic Reviews and Meta-Analyses (PRISMA) flow diagram of study selection.

This systematic review and meta-analysis was registered in the International Prospective Register of Systematic Reviews (PROSPERO; registration number: CRD420251141392) and conducted in accordance with the Preferred Reporting Items for Systematic Reviews and Meta-Analyses (PRISMA) guidelines [26] and the A Measurement Tool to Assess Systematic Reviews version 2 (AMSTAR-2) methodological standards [27] (see Fig. 1). A completed PRISMA 2020 checklist is provided as Supplementary Material (Additional File 1). The last literature search was completed on 28 August 2025. The research question was defined using the PICOS framework to ensure methodological rigour and transparency. Specifically, the study aimed to determine whether children, adolescents and adults fitted with scleral lenses (Population) experience significant changes in CCT, corneal/stromal swelling or IOP (Outcomes) as a result of scleral lens wear (Intervention/Exposure), compared with baseline (pre-lens) values or control/comparator eyes (Comparator). All included studies were prospective, observational, controlled or crossover designs (Study design), and exposures of interest included scleral, mini-scleral or corneo-scleral lenses with varying oxygen transmissibility, vault and design characteristics. Primary outcomes focused on quantifying changes in CCT, percentage of corneal or stromal swelling and IOP, both during scleral lens wear and after lens removal. Secondary analyses explored the potential influence of lens parameters (e.g., Dk/t, vault), patient age, ocular condition (healthy vs. irregular/mixed) and study design on these outcomes, as well as methodological heterogeneity and risk of bias across studies. Through this comprehensive approach, this review sought to synthesise the best available evidence on the short- and longer-term physiological effects of scleral lens wear, identify gaps and inconsistencies in the literature and inform clinical practice and future research priorities in the field of specialty contact lens fitting.

### Eligibility Criteria

Studies were excluded if they met any of the following conditions: case reports, case series, conference abstracts or studies without a comparative or control group; systematic or narrative reviews; duplicate publications from the same dataset; studies rated as high risk of bias or lacking sufficient methodological rigour. Additional exclusions were applied to studies with non-comparable or incomplete demographic data, unclear or inadequate definitions of ocular diagnosis or scleral lens fitting protocols, absence of relevant outcomes related to CCT, corneal swelling or IOP or insufficient statistical data (e.g., missing means, standard deviations or confidence intervals (CIs)) required for quantitative synthesis.

### Information Sources

A comprehensive and systematic literature search was conducted in three major electronic databases: PubMed, Web of Science and Scopus, with no restrictions on publication date or language. To ensure thoroughness and capture all relevant studies, the reference lists of all included articles were also screened manually to identify additional publications that may not have been retrieved through the initial database search.

### Search Methods for Identification of Studies

The search strategy combined both controlled vocabulary and free-text terms related to scleral lens wear and relevant ocular outcomes. Specifically, the following terms were used: (‘scleral lens’ OR ‘scleral lenses’ OR ‘mini-scleral’ OR PROSE) AND (‘corneal thickness’ OR ‘corneal central thickness’ OR CCT OR pachymetry OR ‘corneal edema’ OR ‘corneal oedema’ OR ‘corneal swelling’ OR hypoxia OR hypoxic OR ‘oxygen permeability’ OR ‘oxygen transmissibility’ OR ‘gas permeability’ OR ‘intraocular pressure’ OR IOP). Detailed search strategies for each database are provided in Additional File 2. Three reviewers independently screened titles and abstracts as well as full-text articles for eligibility. Any disagreements were resolved by discussion and consensus. No language restrictions were applied; studies published in languages other than English were translated by the authors using DeepL Translator (DeepL SE, deepl.com) and Google Translate (Google LLC, translate.google.com) when necessary and were included if they reported relevant data. When required, translations were cross-checked by the authors to ensure accuracy.

### Data Extraction and Data Items

Three authors independently extracted data from all eligible studies. For each study included, key characteristics were collected, including the name of the first author, year of publication, country or region, study design, sample size, mean age of participants, underlying ocular condition, scleral lens type and diameter, oxygen transmissibility (Dk/t), central vault and measurement timepoints for outcomes. Any discrepancies in data extraction or study inclusion were resolved through discussion and consensus among the three reviewers. Study record management, including duplicate removal and tracking of eligibility decisions, was performed using Rayyan (Rayyan Systems Inc., rayyan.ai). The primary variables extracted were CCT, corneal oedema or swelling, IOP and related pachymetric or physiological parameters before, during and after scleral lens wear. Corneal oedema was quantified as the percentage change in corneal thickness from baseline, as reported in the original studies and typically derived from pachymetric measurements. Where available, stromal swelling was analysed separately and refers specifically to changes in stromal thickness, whereas corneal swelling reflects changes in total corneal thickness. Additional variables such as region/country, age range, diagnostic methods, lens material, study duration and reporting of relevant confounding factors (e.g., exclusion criteria for corneal pathology or prior surgery) were also recorded to facilitate subgroup analyses and methodological quality assessment.

### Risk of Bias Assessment

The methodological quality and risk of bias of the included observational studies were assessed independently by three reviewers using the Methodological Index for Non-Randomised Studies (MINORS), as developed by Slim et al. [28]. The MINORS tool evaluates key domains such as clarity of study objectives, consecutive inclusion of participants, appropriateness of inclusion criteria, objectivity of outcome assessment and adequacy of follow-up procedures.

For comparative (case-control or cohort) studies, the total MINORS score ranges from 0 to 24, with studies categorised as very low (0–6), low (7–10), moderate (11–15) or high quality (16–24). Although the MINORS instrument also includes a scoring system for non-comparative studies, only comparative studies were eligible for inclusion in this review. Disagreements in quality assessment were resolved through discussion and consensus amongst the three reviewers. The detailed results of the risk of bias assessment are presented in Table 1.

**Table 1:** Assessment of the quality of studies through the Methodological Index for Non-Randomised Studies (MINORS).

Study	Clearly stated aim	Consecutive patients	Prospective collection of data	Endpoints	Assessment endpoint	Follow-up period	Loss less than 5%	Study size	Adequate control group	Contemporary group	Baseline control	Statistical analyses	MINORS
Cheung et al. [31]	2	1	2	2	1	2	2	0	2	2	2	2	20
de Luis Eguileor et al. [32]	2	2	2	2	2	2	2	0	1	2	2	2	21
Fogt et al. [33]	2	2	2	2	2	1	2	0	2	2	2	2	21
Jiang et al. [34]	2	2	2	2	2	1	2	0	1	2	2	2	20
Kramer and Vincent [35]	2	2	2	2	2	2	1	1	1	2	2	2	21
Kumar et al. [36]	2	1	2	2	2	1	2	1	1	2	2	2	20
Lin et al. [37]	2	1	0	2	2	2	2	0	2	2	1	2	18
Litvin et al. [38]	2	2	2	2	2	1	2	2	1	2	2	2	22
Macedo-de-Araújo et al. [39]	2	2	2	2	2	2	1	0	2	2	2	2	21
Michaud et al. [40]	2	2	2	2	1	2	2	0	2	2	2	2	21
Nau et al. [41]	2	2	2	2	2	2	2	2	2	2	2	2	24
Nau et al. [42]	2	2	2	2	2	2	2	1	2	2	2	2	23
Obinwanne et al. [43]	2	2	2	2	2	2	2	1	2	2	2	2	23
Queiruga-Piñeiro et al. [44]	2	2	2	2	2	2	2	2	2	2	2	2	24
Serramito et al. [45]	2	2	2	2	2	2	2	2	0	2	2	2	22
Shahnazi et al. [46]	2	2	0	2	1	2	2	0	0	0	0	2	13
Soeters et al. [18]	2	2	0	2	1	2	2	1	0	0	0	2	14
Tan et al. [47]	2	1	2	2	2	2	2	0	0	0	0	2	13
Tan et al. [48]	2	1	2	2	2	2	2	0	0	0	0	2	13
Vincent et al. [49]	2	1	2	2	2	2	2	0	0	0	0	2	13
Vincent et al. [50]	2	2	2	2	2	2	2	2	2	2	2	2	24
Walker et al. [51]	2	2	2	2	2	2	2	2	2	2	2	2	24

### Assessment of Results

All outcomes of interest (CCT, corneal or stromal swelling and IOP) were treated as continuous variables. For each study, mean differences (MDs) with 95% CIs were calculated by comparing baseline (pre-lens) values with those measured during scleral lens wear or after lens removal. MDs were defined as post-lens values minus baseline; therefore, positive values indicate an increase in the outcome during or after scleral lens wear (e.g., corneal thickening or swelling), whereas negative values indicate a decrease compared to baseline. When necessary to harmonise scales or units across studies, standardised MDs were used. Duration of lens wear was considered through subgroup stratification (e.g., same day, 1 week, 1 month and longer follow-up periods). Measurements obtained during scleral lens wear and after lens removal were analysed separately and were not pooled together in the same analysis.

Statistical heterogeneity among studies was quantified using the *I*² statistic and interpreted as low (<25%), moderate (25–50%) or high (>50%). A fixed-effects model was applied when heterogeneity was absent or negligible (*I*² ≤ 50%), whereas a random-effects model was used in the presence of substantial heterogeneity.

In cases of missing or incomplete data (e.g., absent measures of variance), methodological recommendations from the *Cochrane Handbook for Systematic Reviews of Interventions* [29] were followed, including contacting study authors when feasible. All meta-analyses and figure generation (forest and funnel plots) were performed using Review Manager (RevMan) version 5.4.1 (The Cochrane Collaboration, cochrane.org).

### Publication Bias

Potential publication bias was evaluated for all primary outcomes (CCT during and after scleral lens wear, corneal/stromal swelling and IOP after lens removal) through visual inspection of funnel plots generated in Review Manager (RevMan) version 5.4.1. Symmetry of the plots was interpreted as evidence against publication bias, whereas asymmetry was considered a possible indication of selective reporting or underrepresentation of smaller studies with non-significant results.

### Additional Analyses

Sensitivity analyses were conducted to test the robustness of the pooled results by sequentially removing studies identified as highly influential within each outcome domain (CCT, corneal/stromal swelling and IOP). This approach allowed assessment of the impact of individual studies on overall effect estimates and facilitated a better understanding of potential sources of heterogeneity. All analyses were performed in Review Manager (RevMan) version 5.4.1, applying a random-effects model when significant heterogeneity was detected.

In addition, meta-regression analyses were performed using the *metafor* package in R 4.4.2 (r-project.org) to examine whether between-study differences in oxygen transmissibility (Dk/t), vault, mean age or corneal condition influenced changes in CCT or IOP.

The certainty of evidence for each outcome was graded using the Grading of Recommendations, Assessment, Development and Evaluation (GRADE), considering risk of bias, inconsistency, indirectness, imprecision and publication bias [30]. All assessments were carried out independently by two reviewers, with disagreements resolved by discussion and, when required, consultation with a third author.

## Results

### Study Selection

A total of 442 records were initially retrieved from PubMed (*n* = 117), Web of Science (*n* = 135) and Scopus (*n* = 190) (Fig. 1). After removal of duplicates and screening of titles and abstracts, 287 records were excluded for reasons such as not reporting original data, not evaluating scleral lens wear, not reporting outcomes related to corneal oedema or IOP or being non-comparative studies. Subsequently, 155 full-text articles were assessed for eligibility. Of these, 137 were excluded due to non-comparative data, dissimilar demographics, incomplete data, high risk of bias or lack of shared data. Additionally, four relevant studies were identified through manual review of reference lists. In total, 22 studies met the inclusion criteria and were included in both the qualitative synthesis and meta-analysis [16, 20, 31–50].

### Study Characteristics

Supplementary File 3 summarises the key characteristics of the 22 studies included in this systematic review and meta-analysis, all of which evaluated changes in corneal oedema and/or IOP associated with scleral lens wear across a range of populations and lens designs. The studies were conducted in diverse regions, including Australia, Spain, the USA, China, India, Taiwan, Portugal, Canada, Nigeria and the Netherlands. Study designs were predominantly prospective and observational, with several randomised, crossover or controlled trials and sample sizes ranging from 7 to 50 participants per study. Mean ages of participants varied from young adults (mean ~21 years) to older cohorts, depending on the study population and ocular condition.

Included studies encompassed a broad spectrum of eye conditions, such as healthy controls, keratoconus, post-LASIK ectasia, penetrating keratoplasty, irregular corneas and ocular surface disease, as well as healthy neophytes. A variety of scleral lens types and diameters were evaluated, including fenestrated and non-fenestrated designs, mini-scleral and full scleral lenses and materials with Dk/t values ranging from 85 to over 160 (ISO/Fatt). Vault (central clearance) at baseline typically ranged from 74 to over 400 μm, although the timing of measurement (initial insertion vs. post-settling) varied across studies, with several reporting changes in vault over time. Measurement timepoints varied widely between studies and generally included baseline (pre-insertion), during lens wear (at intervals ranging from minutes to several hours) and post-removal assessments. The majority of studies did not declare conflicts of interest. This heterogeneity in study design, lens parameters and populations was considered in the data synthesis and meta-analytic approach.

### Outcomes

#### Effect of Scleral Lens Wear on CCT With Scleral Lens In Situ

**Fig. 2:**
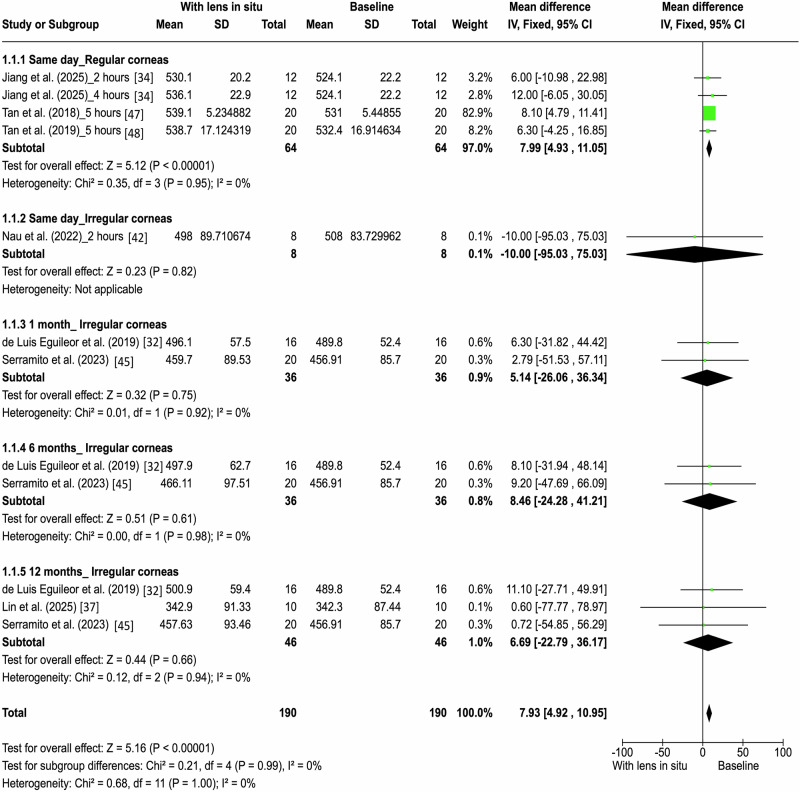
Forest plot of mean differences in central corneal thickness during scleral lens wear. CI confidence interval, IV inverse variance, SD standard deviation.

Overall, changes in CCT during scleral lens wear were small and of limited clinical relevance. Figure 2 shows the pooled analysis of changes in CCT during scleral lens wear. The meta-analysis included seven studies [32, 34, 37, 42, 45, 47, 48], comprising a total of 190 eyes.

For healthy (regular) corneas evaluated on the same day, three studies [34, 47, 48] (*n* = 64 eyes) showed a small but statistically significant increase in CCT with the lens in situ compared to baseline (MD: 7.99 µm; 95% CI: 4.93–11.05; *p* < 0.0001), with no heterogeneity (*I*² = 0%).

In irregular corneas, no significant changes in CCT were observed, either on the same day or after longer-term follow-up (1, 6 and 12 months), and heterogeneity remained negligible across these subgroups (*I*² = 0%).

Overall, the pooled MD was 7.93 µm (95% CI: 4.92–10.95; *p* < 0.0001), indicating a small overall increase in CCT during scleral lens wear. No significant differences were found between subgroups. Although some individual studies showed slight increases in CCT with the lens in situ, these differences were small and unlikely to be clinically meaningful.

#### Effect of Scleral Lens Wear on CCT After Scleral Lens Removal

**Fig. 3:**
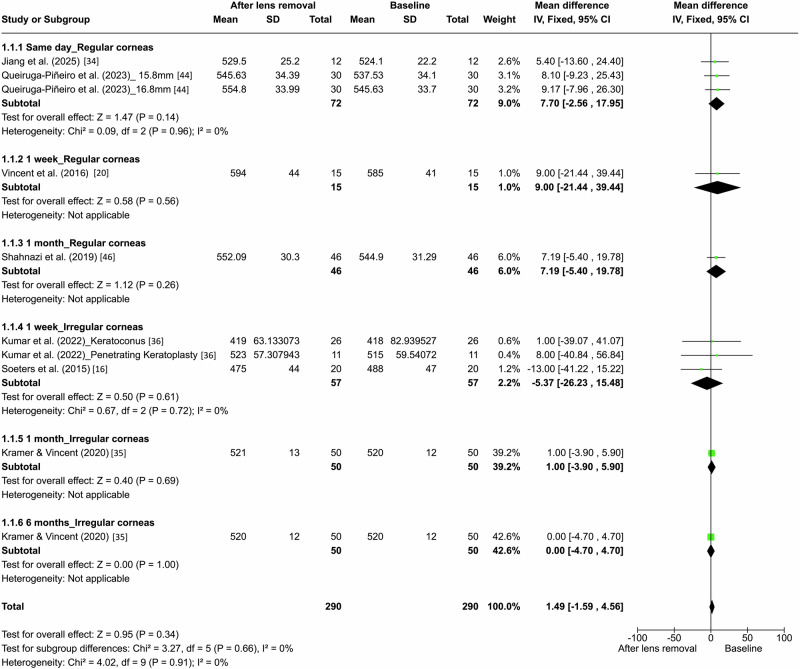
Forest plot of mean differences in central corneal thickness after scleral lens removal. CI confidence interval, SD standard deviation.

Overall, no clinically meaningful changes in CCT were observed after scleral lens removal. Figure 3 presents the pooled analysis of CCT changes after scleral lens removal. The meta-analysis included seven studies [16, 34–36, 44, 46, 49] with a total of 290 eyes.

For healthy corneas, no significant differences were found between baseline and post-removal measurements at any time point. Similarly, in irregular corneas, CCT remained stable across follow-up periods (1 week to 6 months), with CIs consistently crossing zero.

Overall, the pooled MD was 1.49 µm (95% CI: −1.59 to 4.56; *p* = 0.34), indicating no significant change in CCT after lens removal. Heterogeneity was negligible (*I*² = 0%), suggesting consistent findings across studies.

#### Corneal and Stromal Swelling Associated With Scleral Lens Wear

**Fig. 4:**
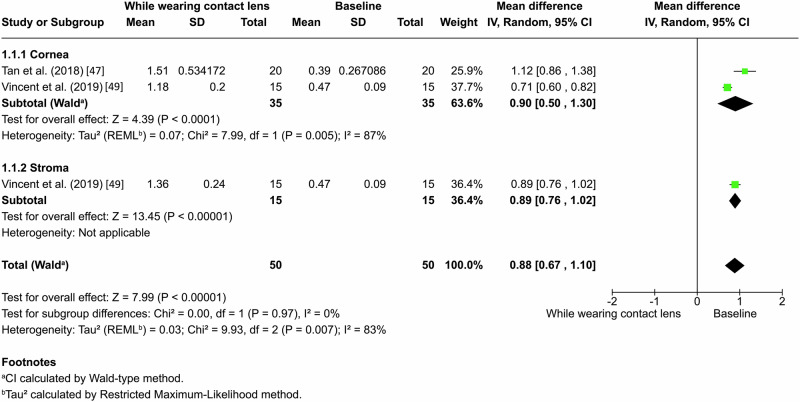
Forest plot of mean differences in corneal and stromal swelling (%) during scleral lens wear. CI confidence interval, SD standard deviation.

Overall, corneal and stromal swelling during scleral lens wear was small in magnitude (≈1%), despite being statistically significant and based on limited evidence. Figure 4 presents the pooled results for corneal and stromal swelling (%) during scleral lens wear, based on data from two studies [47, 49] (*n* = 50 eyes). Both studies reported changes in total corneal swelling, while only one [49] provided data for stromal swelling.

For total corneal swelling, the meta-analysis demonstrated a significant increase in thickness during scleral lens wear compared to baseline, with a pooled MD of 0.90% (95% CI: 0.50–1.30%; *p* < 0.0001). Substantial heterogeneity was observed between studies (*I*² = 87%).

For stromal swelling, only a single study [49] contributed data, reporting an MD of 0.89% (95% CI: 0.76–1.02%; *p* < 0.0001).

The overall pooled MD was 0.88% (95% CI: 0.67–1.10%; *p* < 0.0001), indicating a small but statistically significant increase in both corneal and stromal thickness during scleral lens wear. No significant differences were found between the subgroups (cornea vs. stroma; *p* = 0.97), and overall heterogeneity was moderate (*I*² = 83%).

#### IOP After Scleral Lens Removal

**Fig. 5:**
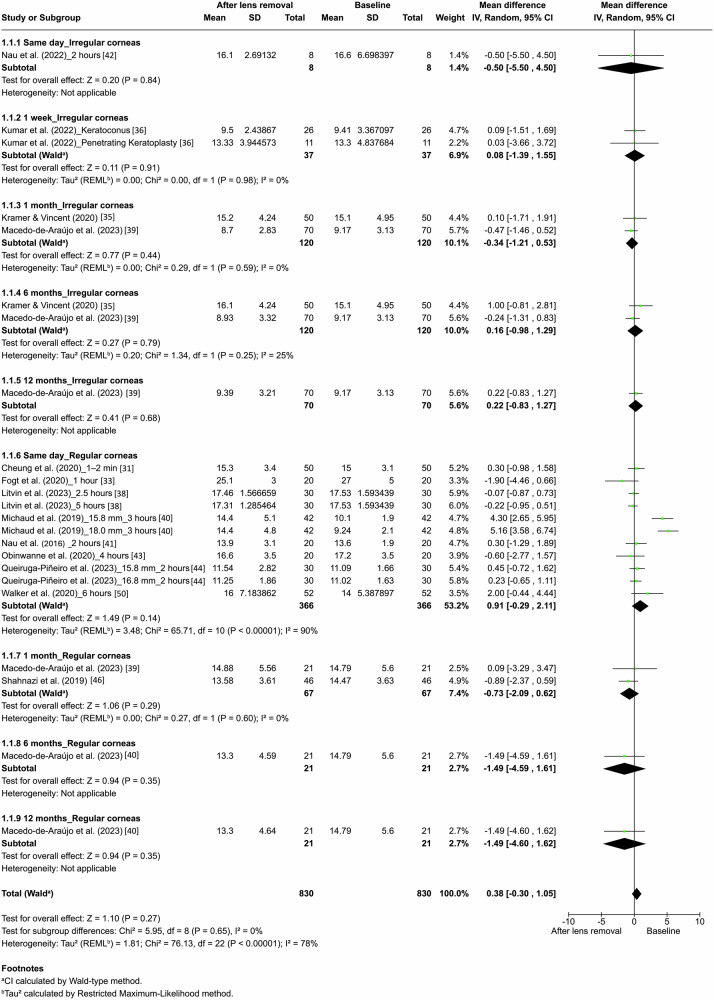
Forest plot of mean differences in intraocular pressure in regular and irregular corneas. CI confidence interval, SD standard deviation.

Overall, no clinically meaningful changes in IOP were observed after scleral lens removal. Figure 5 presents the pooled analysis of changes in IOP after scleral lens removal, based on data from 13 studies [31, 33, 35, 36, 38–44, 46, 50] including a total of 830 eyes at baseline and after lens removal (not individual participants, as several studies reported outcomes per eye).

For regular (healthy) corneas, same-day measurements (*n* = 366 eyes, eight studies [31, 33, 38, 40, 41, 43, 44, 50]) demonstrated no statistically significant change in IOP after lens removal compared to baseline (MD: 0.91 mmHg; 95% CI: −0.29 to 2.11; *p* = 0.14), despite substantial heterogeneity among studies (*I*² = 90%). Results were similar for regular corneas assessed at 1 week (MD: −0.73 mmHg; 95% CI: −2.09 to 0.62; *p* = 0.29) and at later follow-up points, with no significant changes observed.

For irregular corneas, IOP measurements at various follow-up intervals (same day, 1 week, 1 month, 6 months and 12 months) also revealed no statistically significant differences after scleral lens removal compared to baseline, with MDs close to zero and CIs crossing zero at all timepoints, except for a small decrease at 6 and 12 months (MD: −1.49 mmHg; 95% CI: −4.59 to 1.61).

Overall, the pooled MD in IOP after scleral lens removal was 0.38 mmHg (95% CI: −0.30 to 1.05; *p* = 0.27), indicating no statistically significant change in IOP compared to baseline. Subgroup analysis found no significant differences between timepoints or corneal status.

Substantial heterogeneity was observed in the overall analysis (*I*² = 78%), likely reflecting variability in study design, populations, lens parameters and measurement protocols rather than a consistent physiological effect.

### Sensitivity Analysis

**Fig. 6:**
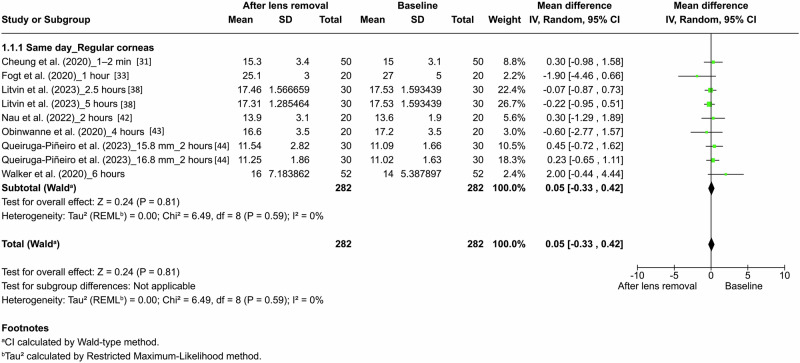
Sensitivity analysis of intraocular pressure after scleral lens removal in regular corneas following exclusion of Michaud et al. [40]. CI confidence interval, SD standard deviation.

A sensitivity analysis was performed to identify and address sources of heterogeneity in IOP outcomes after scleral lens removal in regular corneas (same day). Michaud et al. [40] was identified as a major contributor to heterogeneity in the pooled analysis (Fig. 6). Exclusion of this study resulted in a pooled MD of 0.05 mmHg (95% CI: −0.33 to 0.42), which remained non-significant. Importantly, this adjustment reduced statistical heterogeneity from 90% to 0% (*I*² = 0%).

### Publication Bias

**Fig. 7:**
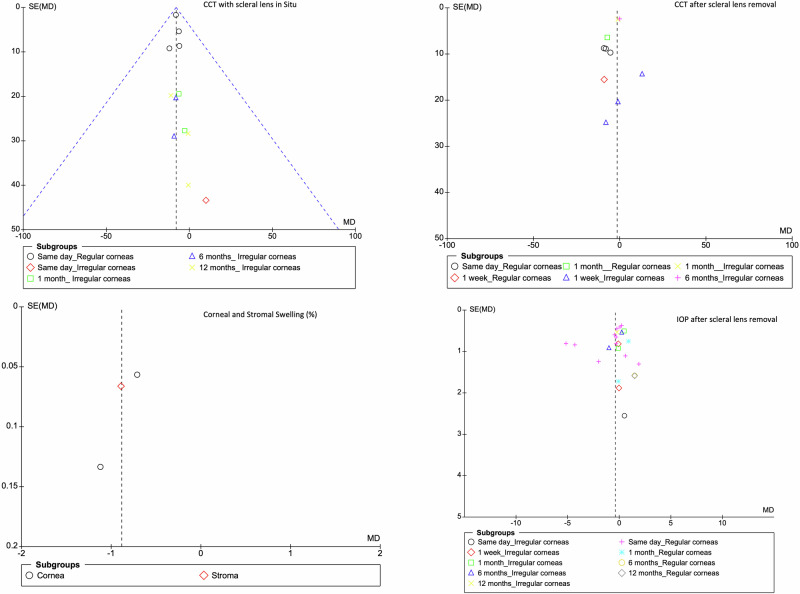
Assessment of publication bias. CCT central corneal thickness, MD mean difference, SE standard error.

Publication bias was assessed using funnel plots for all primary outcome domains in the meta-analysis, including CCT after scleral lens wear, CCT after lens removal, corneal swelling (%) and IOP. As illustrated in Fig. 7, visual inspection of the four funnel plots demonstrated generally symmetrical distributions of study estimates around the pooled MDs, with no evidence of substantial asymmetry for most outcomes.

For both CCT after scleral lens wear and after lens removal, the funnel plots appeared symmetric, indicating a low likelihood of publication bias or small-study effects in these domains. The funnel plot for IOP likewise showed a balanced distribution of studies, supporting the reliability of the pooled estimates.

The funnel plot for corneal swelling (%) displayed a slightly broader spread and minor asymmetry, which may be attributable to variations in study design or sample size. However, no consistent pattern indicative of significant publication bias was detected.

### Meta-regression of Central Corneal Thickness and Intraocular Pressure Outcomes

Meta-regression analyses were performed to evaluate whether oxygen transmissibility (Dk/t), vault, mean age or corneal condition influenced changes in CCT or IOP associated with scleral lens wear. For CCT with the lens in situ, none of the moderators showed a statistically significant association with the observed changes. Specifically, the regression coefficient for Dk/t was 0.001 (95% CI: −2.21 to 2.21, *p* = 0.99), for vault it was 0.02 (95% CI: −0.68 to 0.72, *p* = 0.96) and for mean age −0.09 (95% CI: −16.90 to 16.72, *p* = 0.99). Similarly, after scleral lens removal, Dk/t (−0.17; 95% CI: −1.44 to 1.10; *p* = 0.80), vault (0.01; 95% CI: −0.10 to 0.11; p = 0.88), mean age (0.00; 95% CI: −1.54 to 1.55; *p* > 0.99) and corneal status (5.72; 95% CI: −31.08 to 42.53; *p* = 0.76) were not significantly associated with changes in CCT. For IOP, none of these factors were significantly related to the MD after lens wear, with regression coefficients for Dk/t of 0.07 (95% CI: −0.05 to 0.20; *p* = 0.25), vault −0.001 (95% CI: −0.014 to 0.012; *p* = 0.87), mean age 0.02 (95% CI: −0.10 to 0.13; *p* = 0.79) and for corneal status 2.02 (95% CI: −0.95 to 4.98; *p* = 0.18). Across all models, residual heterogeneity was negligible (*I*² = 0%), indicating high consistency in the results regardless of lens design, patient age or corneal condition.

### GRADE

The GRADE summary of findings for all key outcomes is presented in Table 2. The certainty of evidence for changes in CCT with the scleral lens in situ, as well as for IOP after lens removal, was rated as very low, mainly due to the serious risk of bias arising from non-randomised study designs. For CCT after lens removal and for corneal or stromal swelling, the certainty of evidence was rated as low, primarily due to potential imprecision and limitations in study design.

**Table 2:** Grading of Recommendations, Assessment, Development and Evaluation (GRADE) assessment of the quality of the evidence and the strength of the recommendations.

Certainty assessment							№ of patients		Effect		Certainty	Importance
№ of studies	Study design	Risk of bias	Inconsistency	Indirectness	Imprecision	Other considerations	[intervention]	[comparison]	Relative (95% CI)	Absolute (95% CI)		
Effect of scleral lens wear on central corneal thickness with scleral lens in situ												
6	Non-randomised studies	Serious^a^	Not serious	Not serious	Not serious	None	190/380 (50.0%)	190/380 (50.0%)	**OR −7.93** (−10.95 to −4.92)	**644 more per 1000** (from 601 more to 755 more)		CRITICAL
New effect of scleral lens wear on central corneal thickness after scleral lens removal												
7	Non-randomised studies	Serious^a^	Not serious	Not serious	Not serious	None	290/580 (50.0%)	290/580 (50.0%)	**OR −1.49** (−4.56 to 1.59)	**1000 more per 1000** (from 114 more to 781 more)		CRITICAL
Corneal and stromal swelling associated with scleral lens wear												
2	Non-randomised studies	Serious^a^	Not serious	Not serious	Not serious	None	50/100 (50.0%)	50/100 (50.0%)	**OR −0.88** (−1.10 to -0.67)	**1000 fewer per 1000** (from 1000 fewer to 1000 more)		CRITICAL
New intraocular pressure after scleral lens removal												
14	Non-randomised studies	Serious^a^	Not serious	Not serious	Not serious	None	366/732 (50.0%)	366/732 (50.0%)	**OR −0.38** (−1.05 to 0.30)	**1000 fewer per 1000** (from 269 fewer to 1000 more)		CRITICAL

*CI* confidence interval, *OR* odds ratio.

^a^Lack of randomisation.

No outcomes were supported by high-certainty evidence, and most estimates were based on studies with moderate to high risk of bias, indirectness or imprecision. While the meta-analysis indicates a small increase in CCT during scleral lens wear, with no significant change in CCT after lens removal, minimal stromal swelling and no clinically relevant increase in IOP, the overall certainty in these results is limited. Larger, well-designed randomised controlled trials are necessary to strengthen the evidence base and clarify the ocular effects of scleral lens wear.

## Discussion

This systematic review and meta-analysis demonstrates that scleral lens wear induces small and predominantly transient physiological changes. The study found a small increase in CCT with the lens in situ, no significant difference after lens removal, a low but consistent level of corneal swelling during wear (around 1–2%) and no clinically meaningful increases in IOP. These findings are largely consistent with the recent literature, although some discrepancies emerge that can be explained by differences in lens design, reservoir characteristics, patient populations and measurement protocols.

The pooled data indicated a small but statistically significant increase in CCT while the lens was in place and no significant change after removal. These results can be interpreted alongside previous studies reporting small increases in corneal thickness or oedema during scleral lens wear, such as Vincent et al. [20], who found <2% oedema after 8 h and Esen and Toker [17], who described a slight thickening of about 1.3%. Likewise, Iqbal and Mahadevan [51] observed a modest increase in corneal thickness that resolved by the following morning. Although these studies reported slight increases in thickness, the magnitude of change was small and likely reflects a combination of mild hypoxic effects, lens settling and measurement variability. Taken together, the available evidence suggests that scleral lens wear induces only minor and reversible physiological changes rather than clinically meaningful alterations in corneal structure.

At a microstructural level, the present results are in line with Consejo et al. [52], who showed significant changes in stromal texture parameters after 8 h of lens wear, despite the limited magnitude of overall oedema. This convergence suggests that hypoxic stress may manifest as subtle stromal remodelling that is not always captured by CCT alone. It also highlights the importance of advanced imaging in revealing subclinical changes that complement pachymetric data. By contrast, Lafosse et al. [19] found that corneo-scleral lenses with smaller diameters induced less increase in CCT and lower hypoxic stress than full scleral lenses, even though tear osmolarity remained stable. Although the current analysis did not stratify outcomes by diameter, the direction of this difference is consistent with the conclusion that larger diameters and thicker fluid reservoirs impose greater oxygen demand and can accentuate hypoxic effects.

Further insight is provided by Iqbal et al. [53], who quantified oedema across both central and peripheral corneal regions and reported a slight trend toward greater peripheral swelling. While the current pooled analysis could not stratify consistently by region, the overall low levels of oedema observed in both cases point to the fluid reservoir as the primary barrier to oxygen diffusion. This interpretation is reinforced by Fisher et al. [54], who showed that a single peripheral fenestration did not significantly reduce oedema when Dk/t and reservoir thickness were controlled and by Wang et al. [55], who demonstrated that reservoir thickness decreases exponentially during the first hours of wear but remains asymmetric, typically thicker inferiorly. These observations support the notion that the distribution and magnitude of the reservoir, more than incremental increases in material permeability, govern the oxygen balance under scleral lenses.

Lens geometry adds another layer of explanation. Vincent et al. [6] reported that average thickness across the entire lens, rather than central thickness alone, provides a more reliable representation of oxygen transmissibility. This helps explain why the meta-regressions did not identify a robust association between reported Dk/t and corneal or pressure outcomes: reliance on central thickness as a proxy may over- or under-estimate the true physiological effect, particularly in high-power designs with non-uniform thickness profiles.

This pooled analysis of IOP, based on 14 studies, showed no significant overall change after lens removal, with substantial between-study variability (*I*² = 78%). These findings are consistent with Shahnazi et al. [46], who observed a small increase in CCT but no IOP change in patients with ocular surface disease. The discrepancy with older historical reports, such as Huggert [25], who described marked pressure elevations with glass scleral lenses, is likely explained by the evolution of lens technology. Modern lenses employ oxygen-permeable materials, refined landing zones and reduced scleral compression, while measurement accuracy has also improved with contemporary tonometric methods. The current meta-regressions did not show any significant influence of vault, Dk/t or age on IOP outcomes, suggesting that small fluctuations are multifactorial and likely compensated by normal outflow mechanisms. These results suggest that scleral lenses do not lead to clinically meaningful changes in IOP after lens removal in most patients. However, as IOP was not assessed during lens wear, potential transient fluctuations while the lens is on the eye cannot be excluded, and monitoring remains prudent in high-risk groups such as those with glaucoma, ocular hypertension, high vaults or prolonged wear.

The relevance of patient susceptibility is further supported by experimental evidence from McKay et al. [56], who showed that keratoconus fibroblasts are more vulnerable to hypoxia, with reduced collagen secretion and increased expression of degradative enzymes. This suggests that average effects in pooled data may under-estimate risks in subgroups such as ectatic or post-keratoplasty corneas, where chronic hypoxia may exacerbate underlying pathology. In such cases, minimising reservoir depth and avoiding prolonged or overnight wear are critical safety measures.

From a clinical perspective, the Scleral Lens and Ocular Compliance Evaluation (SCOPE) survey by Schornack et al. [57] revealed that fewer than half of practitioners routinely measure IOP, and only about one-third measure corneal thickness during scleral lens assessments. Given that changes in CCT and IOP were negligible in most cases, routine measurement for all wearers may not be necessary.

This review has several limitations that should be considered when interpreting the findings. First, all included studies were non-randomised, mostly small in sample size and frequently limited to single-centre designs, which reduces the overall certainty of evidence and restricts causal inference. Second, heterogeneity in lens parameters (diameter, thickness, reservoir depth, fenestration), patient characteristics (healthy vs. ectatic vs. ocular surface disease) and measurement protocols (timing of pachymetry, tonometric technique) complicates direct comparability across studies. Third, several potentially important variables such as average lens thickness, three-dimensional reservoir distribution and detailed reporting of lens material Dk were described inconsistently, limiting the scope of meta-regression analyses. Furthermore, the meta-regression models were based on a relatively small number of studies for each moderator, and a substantial proportion of studies could not be included due to missing data. As a result, the CIs for several moderator effects were wide, indicating a high degree of imprecision. Although no significant moderator effects were found, clinically meaningful associations cannot be excluded. Finally, most studies assessed short-term wear under open-eye conditions, so extrapolation to prolonged or overnight use remains uncertain. Accordingly, the overall certainty of evidence as rated by the GRADE framework was very low for all outcomes, reflecting the limitations inherent to the current body of research in this field. These findings should be interpreted as the best available synthesis, but with caution regarding the strength of clinical recommendations.

Despite these limitations, this review provides a comprehensive and up-to-date synthesis of scleral lens-induced changes in CCT, corneal oedema and IOP. Beyond the standard methodological framework of systematic reviews, the inclusion of detailed quantitative analyses, meta-regressions exploring lens-specific factors and sensitivity testing across subgroups enhances the interpretability and clinical relevance of the findings. However, the relatively high heterogeneity observed in corneal swelling outcomes (*I*² = 83%) should be interpreted with caution, as only two studies contributed to this analysis. This precluded meaningful sensitivity analyses and restricts the ability to explore potential sources of heterogeneity. Additionally, some variables pre-specified in the protocol, such as lens family and diameter, could not be analysed systematically due to inconsistent reporting across studies. Importantly, the consistency of results across a broad range of populations, ocular conditions and lens designs supports the generalisability of the conclusions and their applicability to clinical practice.

Future research should prioritise well-designed prospective and randomised trials, with standardised protocols for lens geometry, reservoir characterisation (e.g., wide-angle optical coherence tomography (OCT)) and pachymetric/IOP measurement. Longitudinal studies in vulnerable populations, such as those with keratoconus, post-keratoplasty and ocular surface disease, are needed to determine whether small but repeated hypoxic changes accumulate into long-term risk. Advanced imaging (Scheimpflug, OCT-based biomechanical metrics) and molecular studies of stromal response could clarify the subclinical effects of hypoxia further.

From a clinical perspective, the current evidence suggests that scleral lens wear generally induces only mild and reversible physiological changes. However, because the certainty of evidence remains low and data on long-term wear are limited, clinicians should exercise caution when fitting patients with high vaults or pre-existing ocular pathology. Selective monitoring of corneal thickness and IOP may be considered in such cases, while routine measurement for all wearers appears unnecessary.

## Conclusions

This systematic review and meta-analysis indicates that scleral lens wear induces small and mostly reversible physiological changes. CCT shows a slight increase with the lens in situ, but no significant difference after removal. Corneal swelling remains minimal (about 1%), consistent with mild hypoxia under appropriate fitting. IOP does not exhibit clinically relevant changes. Although the overall certainty of evidence is low due to non-randomised designs and small sample sizes, findings suggest that modern high-Dk scleral lenses are generally safe for daytime wear. Selective monitoring of CCT and IOP is advisable in high-risk patients.

## Supplementary Information

Additional file 1

Additional file 2

Additional file 3

## Data Availability

No datasets were generated or analysed during the current study.
