# Supplementary material for: Corneal and Intraocular Pressure Responses to Scleral Lens Wear: A Meta-Analysis
Source: Ophthalmic Physiol Opt. 2026 Jun 2;46(4):765–78. doi: 10.1007/s44402-026-00110-7 (PMC13395828; doi:10.1007/s44402-026-00110-7)
Supplement: Supplementary file 2 — Additional file 2 [file 44402_2026_110_MOESM2_ESM.docx]

**Supplementary File 2.** Full electronic search strategies

Pubmed
Search: **("scleral lens"[Title/Abstract] OR "scleral lenses"[Title/Abstract] OR "mini-scleral"[Title/Abstract] OR PROSE[Title/Abstract]) AND ( "corneal thickness"[Title/Abstract] OR "corneal central thickness"[Title/Abstract] OR "CCT"[Title/Abstract] OR "pachymetry"[Title/Abstract] OR "corneal edema"[Title/Abstract] OR "corneal oedema"[Title/Abstract] OR "corneal swelling"[Title/Abstract] OR "hypoxia"[Title/Abstract] OR "hypoxic"[Title/Abstract] OR "oxygen permeability"[Title/Abstract] OR "oxygen transmissibility"[Title/Abstract] OR "gas permeability"[Title/Abstract] OR "intraocular pressure"[Title/Abstract] OR "IOP"[Title/Abstract] )**

("scleral lens"[Title/Abstract] OR "scleral lenses"[Title/Abstract] OR "mini-scleral"[Title/Abstract] OR "PROSE"[Title/Abstract]) AND ("corneal thickness"[Title/Abstract] OR "corneal central thickness"[Title/Abstract] OR "CCT"[Title/Abstract] OR "pachymetry"[Title/Abstract] OR "corneal edema"[Title/Abstract] OR "corneal oedema"[Title/Abstract] OR "corneal swelling"[Title/Abstract] OR "hypoxia"[Title/Abstract] OR "hypoxic"[Title/Abstract] OR "oxygen permeability"[Title/Abstract] OR "oxygen transmissibility"[Title/Abstract] OR "gas permeability"[Title/Abstract] OR "intraocular pressure"[Title/Abstract] OR "IOP"[Title/Abstract])

Web of Science

TS=("scleral lens" OR "scleral lenses" OR "mini-scleral" OR PROSE)

AND

TS=("corneal thickness" OR "corneal central thickness" OR CCT OR pachymetry OR "corneal edema" OR "corneal oedema" OR "corneal swelling" OR hypoxia OR hypoxic OR "oxygen permeability" OR "oxygen transmissibility" OR "gas permeability" OR "intraocular pressure" OR IOP)

Scopus

TITLE-ABS-KEY("scleral lens" OR "scleral lenses" OR "mini-scleral" OR PROSE)

AND

TITLE-ABS-KEY("corneal thickness" OR "corneal central thickness" OR CCT OR pachymetry OR "corneal edema" OR "corneal oedema" OR "corneal swelling" OR hypoxia OR hypoxic OR "oxygen permeability" OR "oxygen transmissibility" OR "gas permeability" OR "intraocular pressure" OR IOP)
