# Supplementary material for: Corneal and Intraocular Pressure Responses to Scleral Lens Wear: A Meta-Analysis
Source: Ophthalmic Physiol Opt. 2026 Jun 2;46(4):765–78. doi: 10.1007/s44402-026-00110-7 (PMC13395828; doi:10.1007/s44402-026-00110-7)
Supplement: Supplementary file 3 — Additional file 3 [file 44402_2026_110_MOESM3_ESM.docx]

**Additional file 3.** Baseline characteristics of the 22 included studies.

| **Author (Year)** | **Country** | **Study Design** | **Sample Size** | **Age (years)** | **Eye Condition(s)** | **Lens Type(s)** | **Dk/t (material)** | **Vault (µm)** | **Measurement Timepoint** | **COI** |
| --- | --- | --- | --- | --- | --- | --- | --- | --- | --- | --- |
| Cheung et al. (2020)[6] | Australia | Prospective, experimental | 50 | 23 ± 4 | Healthy | Fenestrated scleral, 16.5 mm | Hexafocon A | NR | Pre, during (in situ), post lens removal | No |
| de Luis Eguileor et al. (2019)[7] | Spain | Prospective, 12 m | 9 | 37.6 ± 12.0 | Irregular cornea (KCN 37.5%, KCN+ICRS 37.5%, DALK 18.8%, PK 6.2%) | Rose K2 XL 14.6 mm, tisilcon A (Menicon Z) | >163 (Menicon Z) | 202 → 142 | Baseline, 1, 6, 12 m, post-removal | No |
| Fogt et al. (2020)[8] | USA | Prospective, crossover | 20 | 29 ± 9 | Healthy | Onefit 2.0 (15.2mm), BostonSight (18.0mm) | Not stated (typical high Dk) | 250–400 (central clearance) | Baseline, post-insertion, 1h wear, post-removal | No |
| Jiang et al. (2025)[9] | China | Prospective | 12 | 25.3 ± 3.8 | Healthy | Non-fenestrated scleral (iComFit, Boston XO) | 100 (Boston XO) | 117.5 ± 32.9 (fluid reservoir after 4h); lens sagittal height 3600–4200 | Baseline, 2h, 4h, 5 min after removal | No |
| Kramer & Vincent (2020)[10] | USA, Australia | Prospective, longitudinal | 32 | 44 ± 3 | Mixed: ectasia (KCN, PMD, post-RK), keratoplasty, ametropia, dry eye | Scleral custom, 16–17.5 mm, Boston XO, sMap3D or EyePrintPRO | Dk: 100 (Boston XO); t: 250–460 µm (mean 314) | Central reservoir: 150–300 (mean 213) | Baseline, 1 month, 6 months (post-removal) | No |
| Kumar et al. (2022)[11] | India/Australia | Prospective, repeated measures | 23 | PK: 23±2; KC: 27±8 | Keratoconus (26), Penetrating Keratoplasty (11) | Keracare, Acculens (nonfenestrated, 15.9–16.4 mm) | Dk 100 (ro ufocon D, CT 250 µm) | 250–300 (clearance) | Pre and post 8h lens wear (CORVIS ST) | No |
| Lin et al. (2025)[12] | Taiwan | Case-control, retrospective, 12 months | 8 | 30.4 ± 6.5 | Keratoconus | SoClear mini-scleral (Brighten Optix), 14–15 mm | NR | ~300 | Baseline, 12 months (wash-out 1 week SL before Pentacam) | No |
| Litvin et al. (2023)[13] | USA | Prospective, randomized | 30 | 28 ± 9.8 | Healthy | Scleral, 15.6mm & 18.0mm (bilateral wear) | Not reported | PoLTT: higher in 18mm | Baseline, during wear (1.25, 2.5, 3.75, 5 h), after removal | NR |
| Macedo-de-Araújo et al. (2023)[14] | Portugal | Prospective, longitudinal, 12 m | 50 | 35.6 ± 9.5 | Irregular corneas (KCN, post-LASIK, PK, etc.); | Senso Mini Sclera 16.4 mm (Boston XO, Procornea) | 100 (ISO/Fatt) | Vault (FR) 429→243 (IC), 455→280 (RC) | Baseline (pre), LDV2 (>60 min), 1, 6, 12 m (post-removal) | No |
| Michaud et al. (2019)[15] | Canada | Prospective, randomized | 21 | 24.7 ± 4.1 | Healthy | Scleral, 15.8 mm & 18.0 mm (1 per eye) | NR | ~325–330 at insertion | Baseline, after 4.5 h wear | No |
| Nau et al. (2016)[16] | USA | Prospective, interventional | 29 | 29 ± 6 | Healthy, neophytes | Jupiter scleral 15 mm | Dk 100 (Boston XO)* | NR | Baseline, post-insertion, 1h, 2h, post-removal | No |
| Nau et al. (2022)[17] | USA | Randomized crossover, pilot | 7 | 61 ± 10 | Corneal irregularity (KC, PMD); OSD (neurotrophic keratitis, GVHD) | Jupiter/Europa (spherical haptic, 18.0–18.3 mm); BostonSight SCLERAL (quadrant-specific, 18.0 mm) | NR | NR | Baseline, after 2 h wear, after removal | NR |
| Obinwanne et al. (2020)[18] | Nigeria | Prospective | 20 | 28.7 ± 4.3 | Healthy | Europa Scleral, Boston XO | NR | 288.1 ± 122 | Pre, 10min, 2h, 4h, 10min post-removal | No |
| Queiruga-Piñeiro et al. (2023)[19] | Spain | Prospective, contralateral eye | 30 | 29 ± 5.6 | Healthy | ICD Flexfit 15.8mm (L1), 16.8mm (L2) | Dk 125 (paflufocon B) | 355 (L1), 353 (L2) | Baseline, durante porte (0,1,2h), post-removal | No |
| Serramito et al. (2023)[20] | Spain | Prospective, longitudinal, 12 m | 20 | 43.3 ± 8 | Post-LASIK | ICD 16.5 mm (Paflufocon D, Lenticon) | Dk 100 (Paflufocon D) | NR | Baseline, 1, 6, 12 months (post-removal) | No |
| Shahnazi et al. (2019)[21] | USA | Retrospective | 25 | NR | Ocular Surface Disease (dry eye, cGVHD, SJS, Sjögren) | Scleral lenses 17.0–18.0 mm | NR | NR | Pre vs post lens wear (immediate) | No |
| Soeters et al. (2015)[22] | Netherlands | Prospective, intervention | 14 | 30 (19–49) | Keratoconus | Full scleral lens (18–22 mm, Boston XO2, XO, Equalens II) | Dk: 85 (Equalens II), 100 (XO), 161 (XO2) (ISO/Fatt) | Central clearance: mostly 0.1–0.5 mm (100–500 µm)* | Directly after removal, ≥1 week after removal | No |
| Tan et al. (2018) [23] | USA | Prospective, randomized, crossover | 10 | 21.0 ± 2.0 | Healthy, neophytes | Scleral, 15.6 mm (hofocon A) | Dk 97 (hofocon A) | 74–543 (PoLTT, baseline) | Baseline, 10, 20, 30, 60, 90, 120, 180, 240, 300 min (corneal thickness + tear film) | No |
| Tan et al. (2019)[24] | USA (California) | Prospective, randomized, crossover | 10 | 22.2 [20.6, 23.8] | Healthy, neophyte | Mini-scleral 15.6 mm (hofocon A), 3 Dk levels (100/140/160 Barrer) | Dk/t: mean 32 [29, 35] hBarrer/cm (range 21–47) | PoLTT: 254 μm (range 145–345, baseline) | Baseline, 10, 20, 30, 60, 90, 120, 180, 240, 300 min | No |
| Vincent et al. (2019)[25] | Australia | Prospective, repeated measures | 15 | 22 ± 1 | Healthy | Sealed miniscleral (ICD™ 16.5 mm, Boston XO, min. 0.30 mm thickness) | 100 (Boston XO, Dk) | 335 ± 46 (initial clearance, range 225–431) | Baseline, 15, 30, 45, 60, 90, 120, 240, 480 min | No |
| Vincent et al. (2023)[26] | Australia | Prospective, controlled | 15 | 22 ± 3 | Healthy, neophytes | ICD™ 16.5 mm miniscleral | Dk 100 (Boston XO, hexafocon A), t = 300 µm | 353 ± 131 (baseline), 258 ± 119 (8h) | Baseline, post-8h (post-removal); diurnal control | No |
| Walker et al. (2020)[27] | USA | Prospective, controlled (contralateral eye) | 26 | 28 ± 3 (23–33) | Healthy, neophytes | Zenlens RC 15.4 mm scleral | Dk 100 (material not stated, Zenlens is typically hexafocon A/Boston XO, t~300 µm) | 221 ± 29 (application), 148 ± 27 (6h) | Baseline, 2h, 6h, post-removal | No |

Boston XO/XO2: high-Dk lens materials; cGVHD: chronic graft-versus-host disease; COI: conflict of interest; CORVIS ST: corneal biomechanics/tomography system; CT: central thickness; DALK: deep anterior lamellar keratoplasty; Dk: oxygen permeability of lens material; Dk/t: oxygen transmissibility (permeability-to-thickness ratio); Equalens II: lens material; EyePrintPRO: custom impression-based scleral lens system; FR: fluid reservoir; GVHD: graft-versus-host disease; h: hours; hofocon A: high-Dk lens material; IC: initial clearance; ICD: Ideal Conformance Design (scleral lens model); ICRS: intrastromal corneal ring segments; in situ: during lens wear; KCN: keratoconus; LDV2: study-specific timepoint (>60 minutes lens wear); Menicon Z: high-Dk lens material; min: minutes; mm: millimeters; µm: micrometers; NR: not reported; OSD: ocular surface disease; paflufocon B/D: high-Dk lens materials; PK: penetrating keratoplasty; PoLTT: post-lens tear thickness; post-insertion: after lens insertion; post-removal: after lens removal; pre: before lens insertion; RC: recessed chamber (or central reservoir); SJS: Stevens–Johnson syndrome; SL: scleral lens; sMap3D: 3D ocular surface mapping system; t: thickness; USA: United States of America; vault: central clearance (measured in µm)
